# Views of general practice staff about the use of a patient‐oriented treatment decision aid in shared decision making for patients with type 2 diabetes: A mixed‐methods study

**DOI:** 10.1111/hex.12586

**Published:** 2017-06-21

**Authors:** Anita Wildeboer, Esther du Pon, Jan Schuling, Flora M. Haaijer‐Ruskamp, Petra Denig

**Affiliations:** ^1^ Department of Clinical Pharmacy andPharmacology University of Groningen University Medical Center Groningen Groningen The Netherlands; ^2^ Utrecht University of Applied Sciences Utrecht The Netherlands; ^3^ Department of General Practice University of Groningen University Medical Center Groningen Groningen The Netherlands

**Keywords:** decision aids, diabetes mellitus type 2, patient‐centered care, primary health care, qualitative research, shared decision making

## Abstract

**Background:**

Decision aids can be used to support shared decision making (SDM). A patient‐oriented treatment decision aid (DA) was developed for type 2 diabetes but its use by general practice staff appeared to be limited.

**Objectives:**

To explore views of practice staff towards SDM and the DA.

**Design:**

A mixed‐methods study within the Dutch PORTDA‐diab trial.

**Setting and participants:**

Included were 17 practices with staff members who were responsible for routine diabetes care and had worked with the DA, and 209 of their patients.

**Methods:**

Interviews were conducted focusing on applicability, usefulness and feasibility of the DA. Interviews were tape‐recorded, transcribed verbatim and subjected to content analysis for identifying and classifying views. Patient‐reported data about the use of the DA were collected. Associations between specific views and use of the DA were tested using Pearson point‐biserial correlation.

**Results:**

The majority of practice staff expressed positive views towards SDM, which was associated with making more use of the DA. Most of the staff expressed that the DA stimulated a two‐way conversation. By using the DA, several became aware of their paternalistic approach. Some staff experienced a conflict with the content of the DA, which was associated with making less use of the DA.

**Conclusions:**

The DA was considered useful by practice staff to support SDM. A positive view towards SDM was a facilitator, whereas experiencing a conflict with the content of the DA was a barrier for making use of the DA.

## 1 INTRODUCTION

An active role of patients with chronic diseases is required to carry out treatment decisions and daily self‐management activities.^1^ A patient‐centred care approach is advocated by models of chronic care and in diabetes guidelines to support patients in their active role.^2^, ^3^ A key component of patient‐centred care is shared decision making (SDM), in which patients are involved in decision making regarding their own care.^4^, ^5^ To support SDM, decision aids (DA) can be used.^6^ DAs are designed to supplement the interaction between patients and health‐care providers by providing evidence‐based information and treatment options to patients. Despite their potential efficacy, ineffective or a lack of use of DAs is observed in practice.^7^, ^8^ This can be caused by negative views about the content and form as well as the perceived usefulness and feasibility to apply the DA in routine practice.^9^ Most research on SDM and the use of DAs in health care has been limited to physicians. In many countries, however, general practice staff—such as nurse practitioners and physician assistants—play an important role in the management of diabetes and other chronic diseases. The role of nurses in SDM needs further exploration.^10^

DAs can be used by patients before consulting a health‐care provider or during encounter with the health‐care provider. Compared to usual care, the use of DAs can induce better informed and values‐based choices, and improves communication between patient and health‐care provider.^6^, ^11^ A DA should be easy to apply in daily practice, since competing demands and time pressure are often reported barriers.^9^, ^12^ Also, health‐care providers must have confidence in the content of the DA, which should provide balanced and up‐to‐date information.^13^, ^14^

Taking these considerations into account and following the recommendations for developing high quality DAs,^15^ a patient‐oriented treatment DA was developed for patients with type 2 diabetes.^16^ Patients and health‐care providers were involved in the development of this DA. The DA was intended to empower patients for shared goal setting and treatment decision making. Its effects were evaluated in Dutch primary care in the PORTDA‐diab (Patient Oriented Treatment Decision Aid diabetes) trial, finding no evidence of relevant improved patient empowerment.^17^ The DA in this study was to be used before and during consultations with the diabetes patients by general practice staff. Although most patients reported they had received the DA before their encounter, it appeared that during the consultation the DA was not used to its full extent.^17^ This should have included discussing the patient's personal risks and possibilities for risk reduction with the practice staff. Only 46% of the patients reported that they had received all these elements as intended. This may in part explain why the DA did not improve patient empowerment for setting treatment goals in the PORTDA‐diab trial.^17^

The aim of this study is to explore views of the practice staff towards SDM and learn their opinions about the DA in this context. We thus want to identify facilitators and barriers for using the DA as intended. These findings can be used to improve further development and implementation of DAs to support SDM. We formulated the following research questions:

1. What are the views of the practice staff towards SDM?
2. What are the views of the practice staff about the applicability and feasibility of the DA and its usefulness for SDM?
3. Are these views associated with the use of the DA to its full extent?

## 2 MATERIALS AND METHODS

### 2.1 Design

**Figure 1:**
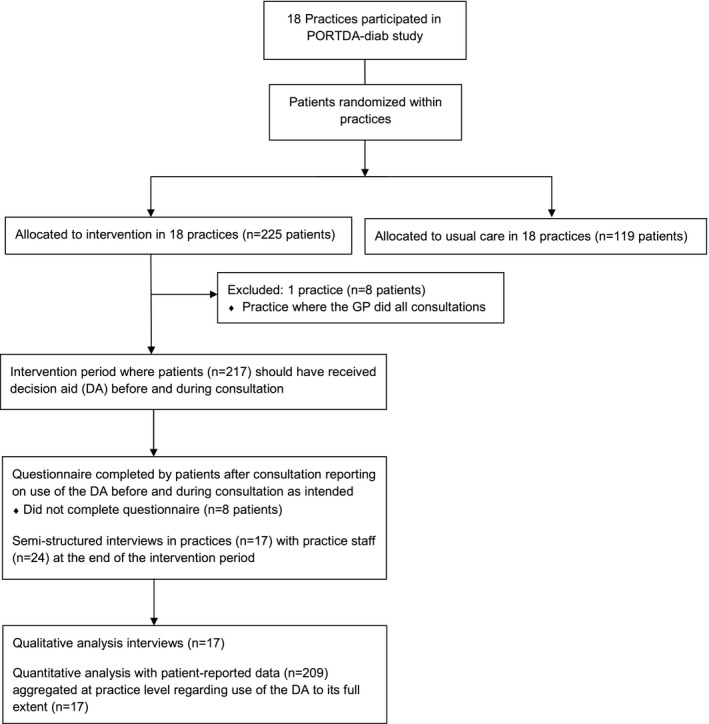
Flow diagram of study participants

This mixed‐methods study was a substudy conducted within the PORTDA‐diab trial.^17^ The PORTDA‐diab trial was a pragmatic randomized controlled trial to assess the effects of the DA on patient empowerment. A patient representative was involved in agreeing the research questions, study design, and interpretation of the results of the PORTDA‐diab trial. Semi‐structured interviews were conducted with the practice staff at the end of the PORTDA‐diab trial in each participating practice (Figure 1). In addition, patient‐reported data were collected about having received all the core elements of the DA as intended.

### 2.2 Decision aid

**Figure 2:**
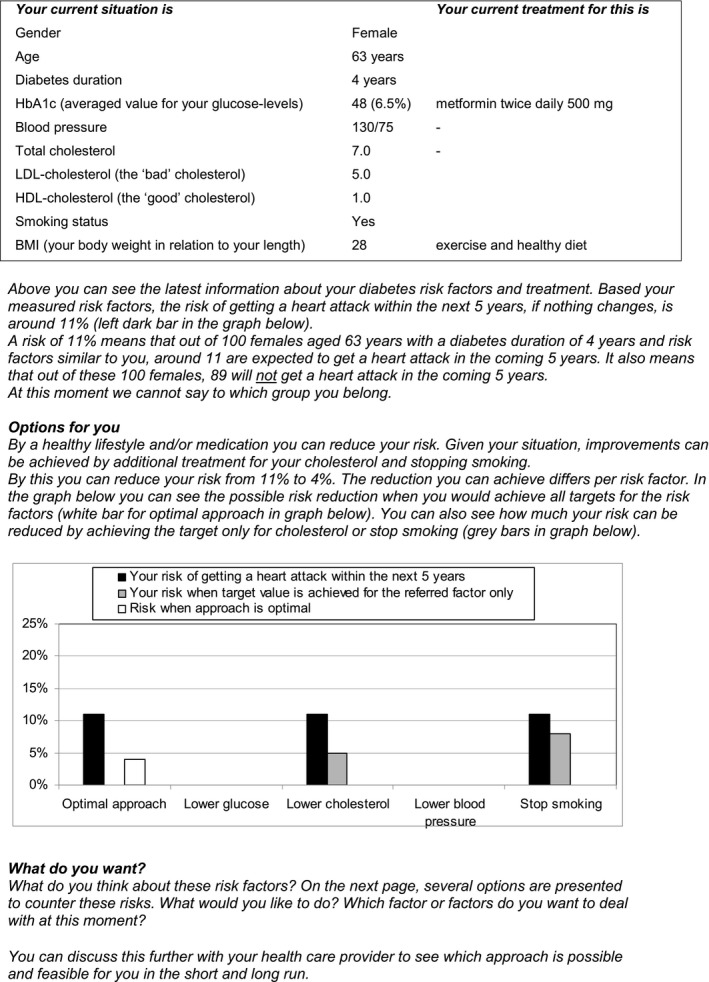
Example showing part of the decision aid

The DA intended to empower patients for shared goal setting and treatment decision making. The DA presented personalized information on risks and treatment for multiple risk factors such as glycosylated haemoglobin, systolic blood pressure, cholesterol and smoking. The DA showed treatment effects on complications, including myocardial infarction risks. Information about the personal risks and potential risk reduction was presented in text and graphs (Figure 2). This was automatically generated via the PORTDA‐diab software from the electronic medical records. Risk information was presented in numbers and words using positive and negative framing, and including an expression of uncertainty. More detailed information about the DA has been published before.^16^ The practice staff had to offer the DA before a routine consultation to consenting patients that had been allocated to the intervention group. The same information was available to the staff, who were instructed to discuss the information with the patients during consultation.

### 2.3 Setting and participants

In the PORTDA‐diab trial, 18 general practices from the Northern region of the Netherlands were recruited by telephone. All practices participated in a regional diabetes disease management programme and have electronic medical record systems that support the use of structured care protocols. Within each practice, patients were randomized to the intervention group receiving the DA or to the control group receiving usual care. Before the study started, all staff had been offered a training course in motivational interviewing and received a training session in risk communication when using the DA. In 17 of the practices, practice staff members were responsible for routine diabetes care and had worked with the DA. All 24 staff members who had worked with the DA were approached by telephone and agreed to participate in the interviews. In one practice, the general practitioner had worked with the DA; this practice was not included in the present study, since our aim was to explore views of practice staff. Of the 217 patients who were randomized to the DA in the 17 practices, 209 completed the questionnaire about having received the core elements of the DA.

### 2.4 Ethics

The medical ethics committee of the University Medical Centre Groningen approved the PORTDA‐diab study (NL29042.042.09). The trial is registered at the Dutch Trial register (NTR1942) with the acronym PORTDA‐diab.

### 2.5 Data collection

Seventeen interviews were conducted by a researcher (male, PhD) at the practice offices. Only the researcher and participants were present. The researcher had been involved in developing and testing the DA. The interviews were held with all staff members who had used the DA. In 11 practices this was one staff member. In the other practices two or three staff members had worked with the DA, who were interviewed together. The interview started with general questions about the practice organization, and the role and level of autonomy of the practice staff in relation to diabetes care. Next, a topic list was used to guide the interviews focusing on the applicability, usefulness and feasibility of the DA in relation to the patient population, SDM and practice organization (Table 1). Also, more general views towards the care process and SDM were retrieved. The topic list was based on previous evaluations of innovation implementations strategies.^18^ Interviews were tape‐recorded and transcribed verbatim. The duration was between one and one and a half hour. No field notes were made during or after the interviews.

**Table 1:** Topic list for the semi‐structured interviews

General aspects	Can you explain how diabetes care is organized in this practice?
	What is your role and responsibility?
	What did you think about the decision aid?
Applicability: content and form	What did you think about the amount of information (text and graphs) in the decision aid?
	What did you think about the information level (degree of difficulty) in the decision aid?
	What did you think about the layout of the decision aid?
	Did you agree with the content of the decision aid?
	Did you experience any difficulties with the decision aid (technical)?
Usefulness: utility and value	How did patients react to the decision aid?
	Did the decision aid support or hinder the conversation with the patients?
	Did the decision aid have an effect on the patient's understanding or insight?
	Did the decision aid have an effect on your own understanding or insight in the patients’ situation?
	Was the decision aid applicable for all patients?
	Did your work process change as you gained more experience with the decision aid?
Feasibility: context	Did working with the decision aid fit within a routine consultation?
	Was it workable to offer the decision aid to the patient before the consultation?
	Was there an adequate place for patients to work with the decision aid prior to the consultation?
	Was it feasible to discuss the decision aid information with the patient?
Other remarks	Do you have any other remarks?

All participating patients were sent a structured questionnaire to report whether they: (i) had received the DA before the consultation, (ii) had discussed their risks for complications with the health‐care provider during the consultation, and how these risks could be reduced. When patients answered positive to these questions, the DA was considered to having been used to ‘its full extent’.^17^

### 2.6 Data analysis

We used qualitative content analysis method, which is a stepwise approach to analyse data content.^19^ A preliminary coding scheme was developed by two researchers with a background in psychology (MSc) and medical sociology (FMH, PhD), respectively, after independently having read the interviews and highlighted codes. Codes are defined as salient or frequently mentioned words, sentences or phrases in the text. After discussing the codes and coming to a consensus, a preliminary coding framework was developed where the codes were placed in themes and subthemes. Next, a specialized diabetes nurse (AW, MSc) and a communication expert (EdP, MSc) applied and refined the coding framework while coding the interviews. All interviews were coded by both researchers. Credibility was established by discussing the framework and coding with a senior researcher (PD, PhD). Discrepancies were solved by discussion and consulting the original transcript information for verification. All codes were documented at practice level, since interviews were conducted per practice.

Next, the results of the qualitative analysis were used to classify practices as supporting or not supporting specific views that could be considered as facilitators or barriers for using a DA to support SDM. This included positive or negative views about involving patients in SDM, and positive or negative views about the DA. Opposing views within one practice were considered as not supporting a specific view. Pearson point‐biserial correlation (IBM SPSS Statistics 24) was used to investigate the associations of binary views with the use of the DA to its full extent at practice level. The latter was defined as the percentage of patients per practice for whom the decision aid was used to its full extent (Table 2).

**Table 2:** Characteristics of the participating practices (n=17)

Practice number	Number of staff included	Profession	Number of patients in control group	Number of patients in intervention group	Number of patients reporting use of decision aid	Percentage of patients reporting use of decision aid to its full extent
1	1	Specialized assistant	6	13	13	38,5%
2	1	Specialized assistant	7	14	14	42,9%
3	3	Specialized assistants	11	23	23	39,1%
4	1	Specialized assistant	2	4	4	75,0%
5	1	Specialized assistant	7	14	13	46,2%
6	1	Specialized assistant	7	10	10	70,0%
7	2	Specialized assistants	10	16	16	37,5%
8	2	Specialized assistants	12	23	22	63,4%
9	1	Specialized assistant	9	19	18	55,6%
10	2	Specialized assistants	10	18	17	23,5%
11	2	Specialized assistants	9	17	16	62,5%
12	1	Specialized assistant	2	4	2	0,0%
13	2	General assistants	1	4	4	50,0%
14	1	General assistant	6	12	12	41,7%
15	1	General assistant	3	4	3	33,3%
16	1	General assistant	4	8	8	62,5%
17	1	Diabetes nurse	8	14	14	35,7%

## 3 RESULTS

### 3.1 Sample characteristics

In 12 of the 17 participating practices, the practice staff consisted of specialized assistants (Table 2). These assistants had received one or two years training in conducting protocol‐based care for chronic diseases, including diabetes. In four other practices, this care was provided by general assistants, who had received only informal training in diabetes care. In one practice, a diabetes nurse conducted diabetes care. Diabetes nurses receive four year general nursing education with an additional one year specialized training in protocol‐based diabetes care. Most practices work with protocols for diabetes care. According to this protocol, diabetes patients visit the practice four times per year. All of the staff conducted routine diabetes consultations, which include physical examination, risk assessment, education and counselling on lifestyle issues. In seven practices, the patients visited the general practitioner for the annual consultation. In most practices, the staff had to consult the general practitioner in case of clinical problems and for changes in medication treatment.

### 3.2 Views towards shared decision making

Seven themes with 12 major (that is, often mentioned) and 6 minor subthemes were identified (Table 3).

**Table 3:** Themes & subthemes for views on shared decision making and decision aid

	Theme	Subthemes
*Views on shared decision making*	Own role in shared decision making	Role is seen as advisory, providing information and education (major)
		Positive towards involving patients in decision making (major)
		Some decisions must be made by provider (minor)
	Patient's role in shared decision making	Patients are not willing or motivated to be involved (major)
*Views on decision aid*	Content and format	Amount is adequate and manageable for patients (major)
		Clear presentation in text and graphs (major)
		Conflict with content, since weight is not included as risk factor (minor)
		Conflict with content, since patients are allowed to decide that they did not want recommended medication (minor)
	Usefulness for provider	Aid increases awareness of own directive or paternalistic approach (major)
		Aid supports to involve patients in actual shared decision making (major)
		Aid is helpful for clarifying risk information (major)
		Aid confirms what they already knew (minor)
	Usefulness for patient	Personalized information is helpful for awareness and motivating patients (major)
		Small risk reductions are not motivating to do anything (major)
	Usefulness for two‐way conversation	Aid supports dialogue with patients and makes them talk and think for themselves (major)
		Aid is helpful to structure consultation and prioritize issues (minor)
	Feasibility and time	Aid fits well in existing practice routines (major)
		Extra time is needed to organize and prepare for the consultation (minor)

#### 3.2.1 Role of practice staff in SDM

In 14 of 17 practices the staff was in general positive about patient participation in decision making and involving patients in conversations and deliberations. They saw their role as advisory and aimed for patient centeredness. They described that they usually provide information and education, talk about benefits and harms of options, and ask what the patients want. Some staff members stated that they always leave the final choice to the patient, even when the patient chooses to do nothing.

Basically what I say is: This is really our advice but you decide for yourself what you want to do or not. … and that you sometimes let people make their own choices. (Practice 11)

In case of medication decisions, the staff of two practices doubted whether they should offer this choice to the patient.

No. I do not know whether you should offer that [drug] choice to the patient. Perhaps it is better to make this choice for them. (Practice 6)

The staff of the remaining three practices did not express a positive view towards involving patients in decision making. One showed a clear paternalistic view by stating that as provider you decide for your patients.

Obviously, they are not used to making the decision themselves because I, as health‐care provider, make this decision for them. (Practice 12)

#### 3.2.2 Role of the patients in SDM

In seven practices, the staff felt that their patients were usually passive, not willing to decide for themselves, and were inclined to shift the responsibility to the health‐care provider. Some staff felt that their patients were not motivated or willing to make decisions because they trivialize the problem.

Then they look at you quizzically, like, what should I do? And, yes, no, you know what is best? (Practice 16)

In such cases, patients did not want to discuss it any further.. because they downplay it a lot. (Practice 1)

### 3.3 Views about the decision aid

#### 3.3.1 Applicability

The amount of information in the DA was considered adequate and manageable for patients according to the staff in 11 of the practices. In the other practices, there were concerns that for some patients it might have been too much information at once. The presented information, both the text and graphs, was easy to understand for patients according to most staff. A few patients needed explanation about the graphs, especially in case of small risk reductions.

In five practices, the staff mentioned that the patient's weight was not included as risk factor in the DA and that they considered this as a missed opportunity. For three of them, this led to a conflict with the DA since they saw reducing overweight as an important target in their population. One other practice experienced a conflict with the DA because patients were allowed to decide that they did not want medication, whereas the staff felt that some drugs, such as statins, were always recommended in patients with type 2 diabetes.

Therefore it frustrated my advice more or less when someone had to lose weight. (Practice 10)

#### 3.3.2 Usefulness

In 14 practices, the staff became more aware of their directive approach, but also of their assumptions about what patients understand or are willing and able to do for themselves. Working with the DA supported them in actual SDM. Some realized that they may have put patients in a passive role. Several experienced how difficult it was to change their approach towards patients.

It made me more conscious of the patient's responsibility. It was already there but now it became stronger. Like: It is your choice, these treatments. (Practice 5)

It is so difficult to let people speak and not assume what they want; to let them make their own choices. (Practice 7)

The DA was appreciated because it presented personalized information clearly laid out for the patients.

I noticed that patients liked it to receive things on paper that are applicable only for them. (Practice 2)

The information provided by the DA was considered not that helpful when it showed little gain in the patient's risk reduction. The staff in nine practices felt that sometimes the risk reductions were so small that it was not motivating for the patients or the staff themselves to do anything.

When the risk reduction is low, it does not tell people that much. … Also, when somebody was young, say below 50, he did not have that much risk anyway (Practice 10)

I think in general, yeah, that the risk reduction is disappointing to me. So, in that sense, not that motivating to start. (Practice 8)

In eight practices, the staff thought that most of their patients were well regulated, except for the ones being overweight. Some staff mentioned that the information from the DA confirmed that they did a good job, whereas another mentioned that it often did not bring any news. In nine practices, the staff mentioned that the DA finally motivated several patients to come into action. Some patients perceived the information as confronting or became scared by the information.

They suddenly saw something like: Hey, now I am seriously going to think about whether I should quit smoking, because this is actually quite different. (Practice 8)

Yeah, some people did want to start using a certain drug or to lower their blood pressure a bit further. (Practice 9)

The DA was considered helpful in 13 practices to make patients more aware of their condition and their personal risks. The staff also found the DA helpful for clarifying risk information.

The decision aid is quite good to make the diabetic aware of his own illness and to make them think for themselves. (Practice 12)

I could explain it clearly, like, if you are, for example, at 10%, and by doing this or that you can change this and reduce this. (Practice 17)

In 15 practices, the staff expressed that the conversation and two‐way exchange with the patient had been supported by using the DA. The DA also helped to structure the consultation and prioritize issues with the patient.

It is also a good way to start a dialogue with patients. (Practice 4)

They come forward themselves, it is more initiated by the patient, since he sees it. They started talking about things, you can nicely discuss it further. (Practice 2)

Like: I'd like to discuss a few things but what do you want to discuss and with what shall we start? (Practice 11)

#### 3.3.3 Feasibility

Presenting the DA prior to consultation and using the DA during consultation fitted well with the existing practice routines but some of the staff mentioned organizational issues. In a few practices it was a challenge to find a quiet or private place for the patients to read the DA. In five practices, it was mentioned that some extra time was needed to organize and prepare things. In two practices, staff reported that they had experienced time pressure during a consultation when a patient became concerned or needed more explanation after reading the information provided by the DA. Most did not mention time constraints during the consultation, and in five cases the staff stated that they were positively surprised that it did not take more time than usual.

For some patients it led to a lot of questions and when they started asking things, I was thinking, yeah, I do not have that much time. (Practice 16)

In terms of time, it turned out better than expected,… In the beginning I thought: ‘Oh, how much consultation time will this take but it turned out well. (Practice 3)

### 3.4 Relation with use of the decision aid to its full extent

Within practices no opposing views were observed. The percentage of patients for whom the DA was used to its full extent was on average 46% and ranged from 0% to 75% (Table 2). Practices expressing positive views towards involving patients in decision making showed a higher use of the DA to its full extent (Table 4). A negative view towards the expected willingness of patients for SDM was not associated with less extended use of the DA. Expressing negative views regarding the content of the DA was associated with less use of the DA to its full extent. Views about the usefulness of the DA or the time needed were not associated with the use of the DA (Table 4).

**Table 4:** Relationship of views with the use of the decision aid to its full extent

Views towards shared decision making and the decision aid	Number of practices		Percentage of patients with use of decision aid to full extent	Correlation with use to full extent	*P*‐value
Positive view towards involving patients in decision making	Yes	14	50%	0.535	0.027
	No	3	25%		
Negative view towards expected willingness of patients to be involved	Yes	7	46%	0.021	0.937
	No	10	45%		
Negative view/conflict with content of decision aid	Yes	4	25%	‐0.641	0.006
	No	13	52%		
Positive view about usefulness of decision aid for health‐care provider	Yes	14	46%	0.026	0.921
	No	3	42%		
Positive view about usefulness of decision aid for patient	Yes	13	44%	‐0.129	0.622
	No	4	50%		
Positive view about usefulness of decision aid for two‐way conversation	Yes	15	48%	0.286	0.266
	No	2	32%		
Negative view about time needed to organize and prepare things	Yes	5	39%	‐0.253	0.327
	No	12	49%		

## 4 DISCUSSION

We found that the majority of general practice staff in our study expressed a positive view towards SDM but that some felt that their patients were not willing or motivated to be involved in the decision making. Most of the staff had positive views about the usefulness of the DA to stimulate actual SDM. On the other hand, some staff members experienced a conflict with the content of the DA. On average, the DA was used to its full extent in 46% of the patients. A positive view towards SDM was associated with making more use of the DA as intended, whereas a negative view towards the content of the DA was associated with making less use of the DA as intended.

Skilled staff with a positive attitude, patients willing to participate and a context which supports SDM can facilitate the SDM process.^20^ The practice staff in general expressed a positive view towards active engagement of patients, which is an important element of SDM.^21^, ^22^ Some of the staff, however, viewed their patients as passive and not willing or motivated to come into action. This pessimism among practice staff has been observed before, especially in relation to lifestyle changes.^23^ By using the DA, several staff members became aware that they were used to taking a directive or paternalistic role, thereby putting patients in a passive role. This is in line with findings from the United States, where decisions made in primary care did not reflect a high level of SDM.^24^ SDM is still not routinely implemented in diabetes care.^25^

Previously, DAs that were developed to support patient involvement in treatment decisions have shown to improve the patients’ decisional comfort.^26^, ^27^, ^28^ It was also found that such DAs can increase patient involvement in a conversation about medication.^26^ Also in our study, the staff perceived that the DA supported more two‐way conversation with the patients. Some of the staff noticed that patients became more active in terms of involvement and also in taking action. It is expected that diabetes patients who are actively involved in decision making are more likely to agree with their provider regarding treatment goals and strategies.^29^ In other cases, patients still wanted the health‐care provider to decide, which has been recognized as ‘welcomed paternalism’.^30^ No specific sociodemographic characteristics were mentioned in relation to getting patients involved in SDM or the usefulness of the DA. This is consistent with a recent meta‐analysis that showed that DAs are effective across diverse sociodemographic patient groups.^31^

Most of the staff was positive about the amount and clarity of the information provided by the DA. The DA was developed following the recommendations for high quality DAs,^15^ including balanced and up‐to‐date information.^13^, ^14^ Several staff members mentioned that the personalized information was particularly appreciated by the patients. It is important to know how the information contained in the DA functioned in the conversation.^32^ Regarding the content of the information, some of the practice staff expressed negative views. Most staff members were trained to follow protocol‐based care, and some experienced a conflict between the DA and their protocol‐based care. The DA did not show the effects of weight reduction since it was based on the UKPDS (United Kingdom Prospective Diabetes Study) risk score that does not include weight.^16^ The practice staff, on the other hand, was used to discuss lifestyle and weight control with patients that were overweight. By not explicitly addressing weight reduction, the DA did not support the advice they wanted to give. Also, the DA offered the option of doing nothing, whereas protocol‐based care may recommend treatment. For health‐care providers following guidelines it may be difficult to allow patients to make decisions based on their own preferences.^33^ It is important that practice staff learn how to integrate patient values and clinical expertise with research evidence.^20^

Some staff seemed surprised or even disappointed about the minor risk reductions presented by the DA. It may be that they had insufficient knowledge about the risk scores. It has been found that specialized nurses overestimate cardiovascular risk.^34^ Disappointment occurred especially when a patient decided to do nothing because of the low risk reduction. The question arises to what extent some of the practice staff wanted to use the DA as a tool to persuade patients to accept protocol‐based care instead of making shared decisions. Such persuader roles in decision making and DA use have previously been identified for physicians.^35^

In general, the use of the DA fitted well within current practice. We did not observe a relation between perceived time constraints and the use of the DA. Previous research reported time restrictions and other barriers for implementing SDM.^9^, ^36^ Most research, however, was limited to physicians who may have less time for a routine consultation than the general practice staff in our study. DAs may lead to shorter as well as longer consultations with an estimated median of 2.55 minutes longer.^6^ For practice staff consultations, with an average duration 21 minutes, this may be perceived as less problematic compared with 10 minutes for a physician visit.^37^ According to several staff members, many of the participating patients were already well regulated. In such cases, some staff found the DA not useful, confirming previous findings.^17^ Others still perceived the DA as helpful because it showed that they were doing it right. So far, not much is known about the usefulness of such affirmative feedback in diabetes management.

The DA was not used to its full extent in more than half of the patients. This problem was also observed in another practice‐based primary care study, where on average 66% of relevant elements were completed by physicians using the treatment DA.^26^ In clinical encounters, DAs become flexible tools used in various ways fitting with both the patients’ and health‐care providers’ roles in decision making.^35^ We observed that not having positive views about SDM was associated with making less use of the DA. Also, having negative views about the content of the DA was associated with making less use of the DA, which confirms previous findings.^7^ Furthermore, we already established that the DA was less used by the staff in patients where little risk reduction was possible.^17^ Aside from these influences of the practice staff, patients may also discourage to apply the DA because they do not want to participate in decision making.^38^ We found that, according to the staff, several patients were not motivated to become involved. It is therefore important that practice staff working with DAs receive training on how to engage patients but also to refrain from making assumptions about the patients’ desired role.^38^

This is a first study in which the views of general practice staff about the use of a patient‐oriented DA in SDM are investigated. Our framework of codes and (sub)themes was carefully built by researchers with different backgrounds, and the coding was conducted independently by two researchers. The qualitative analysis was validated by the senior researcher (PD) by checking the original transcripts. Participants did not provide feedback on the findings. In six practices, two or three staff members were interviewed together. It is possible that the views of some individuals were not fully expressed during these small group interviews. Furthermore, since all practices participated in the PORTDA‐diab trial, the views towards SDM and the use of a DA may be more positive in comparison to the general population of health‐care providers. The quantitative analysis was limited by the small number of practices.

## 5 CONCLUSION

According to the practice staff, the DA stimulated two‐way conversation with the patients and also activated some of the patients. Having a positive view towards SDM appeared to be a facilitator for making use of the DA. On the other hand, experiencing a conflict between the content of the DA and the own protocol‐based views seemed to be a barrier for making use of the DA. More research is needed to investigate the extent to which general practice staff has the intention and ability to apply SDM, taking protocol deviations into account.

## CONFLICT of INTEREST

All authors declare no conflicts of interest.

## References

[1] Powers MA , Bardsley J , Cypress M , et al. Diabetes self‐management education and support in type 2 diabetes: a joint position statement of the american diabetes association, the american association of diabetes educators, and the academy of nutrition and dietetics. Clin Diabetes. 2016;34:70‐80. 27092016 10.2337/diaclin.34.2.70 PMC4833481

[2] Inzucchi SE , Bergenstal RM , Buse JB , et al. Management of hyperglycaemia in type 2 diabetes, 2015: a patient‐centred approach. update to a position statement of the american diabetes association and the european association for the study of diabetes. Diabetologia. 2015;58:429‐442. 25583541 10.1007/s00125-014-3460-0

[3] Wagner EH , Austin BT , Davis C , Hindmarsh M , Schaefer J , Bonomi A . Improving chronic illness care: translating evidence into action. Health Aff (Millwood). 2001;20:64‐78. 11816692 10.1377/hlthaff.20.6.64

[4] Charles C , Gafni A , Whelan T . Decision‐making in the physician‐patient encounter: revisiting the shared treatment decision‐making model. Soc Sci Med. 1999;49:651‐661. 10452420 10.1016/s0277-9536(99)00145-8

[5] Stiggelbout AM , Van der Weijden T , De Wit MP , et al. Shared decision making: really putting patients at the centre of healthcare. BMJ. 2012;344:e256. 22286508 10.1136/bmj.e256

[6] Stacey D , Legare F , Col NF , et al. Decision aids for people facing health treatment or screening decisions. Cochrane Database Syst Rev 2014;(4):CD001431. 24470076 10.1002/14651858.CD001431.pub4

[7] Elwyn G , Scholl I , Tietbohl C , et al. “Many miles to go..”: a systematic review of the implementation of patient decision support interventions into routine clinical practice. BMC Med Inform Decis Mak. 2013;13(Suppl 2):S14. 24625083 10.1186/1472-6947-13-S2-S14 PMC4044318

[8] Wyatt KD , Branda ME , Anderson RT , et al. Peering into the black box: a meta‐analysis of how clinicians use decision aids during clinical encounters. Implement Sci. 2014;9:26. 24559190 10.1186/1748-5908-9-26 PMC3936841

[9] Legare F , Ratte S , Gravel K , Graham ID . Barriers and facilitators to implementing shared decision‐making in clinical practice: update of a systematic review of health professionals’ perceptions. Patient Educ Couns. 2008;73:526‐535. 18752915 10.1016/j.pec.2008.07.018

[10] Clark NM , Nelson BW , Valerio MA , Gong ZM , Taylor‐Fishwick JC , Fletcher M . Consideration of shared decision making in nursing: a review of clinicians’ perceptions and interventions. Open Nurs J. 2009;3:65‐75. 19855848 10.2174/1874434600903010065 PMC2765030

[11] Sepucha KR , Borkhoff CM , Lally J , et al. Establishing the effectiveness of patient decision aids: key constructs and measurement instruments. BMC Med Inform Decis Mak. 2013;13(Suppl 2):S12. 24625035 10.1186/1472-6947-13-S2-S12 PMC4044563

[12] Joseph‐Williams N , Elwyn G , Edwards A . Knowledge is not power for patients: a systematic review and thematic synthesis of patient‐reported barriers and facilitators to shared decision making. Patient Educ Couns. 2014;94:291‐309. 24305642 10.1016/j.pec.2013.10.031

[13] Abhyankar P , Volk RJ , Blumenthal‐Barby J , et al. Balancing the presentation of information and options in patient decision aids: an updated review. BMC Med Inform Decis Mak. 2013;13(Suppl 2):S6. 10.1186/1472-6947-13-S2-S6 PMC4044010 24625214

[14] Agoritsas T , Heen AF , Brandt L , et al. Decision aids that really promote shared decision making: the pace quickens. BMJ. 2015;350:g7624. 25670178 10.1136/bmj.g7624 PMC4707568

[15] Volk RJ , Llewellyn‐Thomas H , Stacey D , Elwyn G . Ten years of the international patient decision aid standards collaboration: evolution of the core dimensions for assessing the quality of patient decision aids. BMC Med Inform Decis Mak. 2013;13(Suppl 2):S1. 10.1186/1472-6947-13-S2-S1 PMC4044280 24624947

[16] Denig P , Dun M , Schuling J , Haaijer‐Ruskamp FM , Voorham J . The effect of a patient‐oriented treatment decision aid for risk factor management in patients with diabetes (PORTDA‐diab): study protocol for a randomised controlled trial. Trials. 2012;13:219. 23171524 10.1186/1745-6215-13-219 PMC3561233

[17] Denig P , Schuling J , Haaijer‐Ruskamp F , Voorham J . Effects of a patient oriented decision aid for prioritising treatment goals in diabetes: pragmatic randomised controlled trial. BMJ. 2014;349:g5651. 25255799 10.1136/bmj.g5651

[18] Peters MAJ , Harmsen M , Laurant MGH , Wensing M . Ruimte voor verandering? knelpunten en mogelijkheden voor verbeteringen in de patiëntenzorg. NijmegenUitgave: Afdeling Kwaliteit van zorg (WOK), UMC St Radboud; 2003.

[19] Saldaña J The coding manual for qualitative researchers (second edition). ISBN‐13: 978‐1446247372. London, UK SAGE Publications Ltd; 2013

[20] Friesen‐Storms JH , Bours GJ , van der Weijden T , Beurskens AJ . Shared decision making in chronic care in the context of evidence based practice in nursing. Int J Nurs Stud. 2015;52:393‐402. 25059684 10.1016/j.ijnurstu.2014.06.012

[21] Makoul G , Clayman ML . An integrative model of shared decision making in medical encounters. Patient Educ Couns. 2006;60:301‐312. 16051459 10.1016/j.pec.2005.06.010

[22] Lewis KB , Stacey D , Squires JE , Carroll S . Shared decision‐making models acknowledging an interprofessional approach: a theory analysis to inform nursing practice. Res Theory Nurs Pract. 2016;30:26‐43. 27024998 10.1891/1541-6577.30.1.26

[23] Jallinoja P , Absetz P , Kuronen R , et al. The dilemma of patient responsibility for lifestyle change: perceptions among primary care physicians and nurses. Scand J Prim Health Care. 2007;25:244‐249. 17934984 10.1080/02813430701691778 PMC3379767

[24] Fowler FJ Jr , Gerstein BS , Barry MJ . How patient centered are medical decisions?: results of a national survey. JAMA Intern Med. 2013;173:1215‐1221. 23712194 10.1001/jamainternmed.2013.6172

[25] Rodriguez‐Gutierrez R , Gionfriddo MR , Ospina NS , et al. Shared decision making in endocrinology: present and future directions. Lancet Diabetes Endocrinol. 2016;4:706‐716. 26915314 10.1016/S2213-8587(15)00468-4

[26] Branda ME , LeBlanc A , Shah ND , et al. Shared decision making for patients with type 2 diabetes: a randomized trial in primary care. BMC Health Serv Res. 2013;13:301. 23927490 10.1186/1472-6963-13-301 PMC3751736

[27] Mann DM , Ponieman D , Montori VM , et al. The Statin Choice decision aid in primary care: a randomized trial. Patient Educ Couns. 2010;80:138‐140. 19959322 10.1016/j.pec.2009.10.008

[28] Mullan RJ , Montori VM , Shah ND , et al. The diabetes mellitus medication choice decision aid: a randomized trial. Arch Intern Med. 2009;169:1560‐1568. 19786674 10.1001/archinternmed.2009.293

[29] Heisler M , Vijan S , Anderson RM , Ubel PA , Bernstein SJ , Hofer TP . When do patients and their physicians agree on diabetes treatment goals and strategies, and what difference does it make? J Gen Intern Med. 2003;18:893‐902. 14687274 10.1046/j.1525-1497.2003.21132.x PMC1494939

[30] Moser A , van der Bruggen H , Widdershoven G . Competency in shaping one's life: autonomy of people with type 2 diabetes mellitus in a nurse‐led, shared‐care setting; a qualitative study. Int J Nurs Stud. 2006;43:417‐427. 16112674 10.1016/j.ijnurstu.2005.06.003

[31] Coylewright M , Branda M , Inselman JW , et al. Impact of sociodemographic patient characteristics on the efficacy of decision AIDS: a patient‐level meta‐analysis of 7 randomized trials. Circ Cardiovasc Qual Outcomes. 2014;7:360‐367. 24823953 10.1161/HCQ.0000000000000006

[32] Hargraves I , Montori VM . Decision aids, empowerment, and shared decision making. BMJ. 2014;349:g5811. 25255800 10.1136/bmj.g5811

[33] Montori VM , Brito JP , Murad MH . The optimal practice of evidence‐based medicine: incorporating patient preferences in practice guidelines. JAMA. 2013;310:2503‐2504. 24165826 10.1001/jama.2013.281422

[34] Scholte op Reimer WJ , Moons P , De Geest S , et al. Cardiovascular risk estimation by professionally active cardiovascular nurses: results from the basel 2005 nurses cohort. Eur J Cardiovasc Nurs. 2006;5:258‐263. 16908217 10.1016/j.ejcnurse.2006.06.007

[35] Tiedje K , Shippee ND , Johnson AM , et al. ‘They leave at least believing they had a part in the discussion’: understanding decision aid use and patient‐clinician decision‐making through qualitative research. Patient Educ Couns. 2013;93:86‐94. 23598292 10.1016/j.pec.2013.03.013 PMC3759553

[36] Legare F , Thompson‐Leduc P . Twelve myths about shared decision making. Patient Educ Couns. 2014;96:281‐286. 25034637 10.1016/j.pec.2014.06.014

[37] Houweling ST , Kleefstra N , van Hateren KJ , et al. Can diabetes management be safely transferred to practice nurses in a primary care setting? A randomised controlled trial. J Clin Nurs. 2011;20:1264‐1272. 21401764 10.1111/j.1365-2702.2010.03562.x

[38] Politi MC , Dizon DS , Frosch DL , et al. Importance of clarifying patients’ desired role in shared decision making to match their level of engagement with their preferences. BMJ. 2013;347:f7066. 24297974 10.1136/bmj.f7066

